# Application of Artificial Intelligence in COVID-19 Pandemic: Bibliometric Analysis

**DOI:** 10.3390/healthcare9040441

**Published:** 2021-04-09

**Authors:** Md. Mohaimenul Islam, Tahmina Nasrin Poly, Belal Alsinglawi, Li-Fong Lin, Shuo-Chen Chien, Ju-Chi Liu, Wen-Shan Jian

**Affiliations:** 1 Graduate Institute of Biomedical Informatics, College of Medical Science and Technology, Taipei Medical University, Taipei 110, Taiwan; d610106004@tmu.edu.tw (M.M.I.); tahmina6969@gmail.com (T.N.P.); alanjian@gmail.com (S.-C.C.); 2 International Center for Health Information Technology (ICHIT), Taipei Medical University, Taipei 110, Taiwan; 3 Research Center of Big Data and Meta-Analysis, Wan Fang Hospital, Taipei Medical University, Taipei 116, Taiwan; 4 Department of Bigdata and Mathmatics, Western Sydney University, Penrith, NSW 2751, Australia; b.alsinglawi@gmail.com; 5 School of Gerontology Health Management, College of Nursing, Taipei Medical University, Taipei 110, Taiwan; fong930@tmu.edu.tw; 6 Department of Physical Medicine and Rehabilitation, Shuang Ho Hospital, Taipei Medical University, New Taipei 235, Taiwan; 7 Neuroscience Research Center, Taipei Medical University, Taipei 110, Taiwan; 8 Professional Master Program in Medicine, Research Center for Artificial Intelligence in Medicine, Taipei Medical University, Taipei City 110, Taiwan; 9 Division of Cardiology, Department of Internal Medicine, Taipei Medical University—Shuang Ho Hospital, Taipei 235, Taiwan; liumdcv@tmu.edu.tw; 10 Research Center for Biomedical Devices Center for Cell Therapy and Regeneration Medicine Taipei Heart Institute (THI), Taipei Medical University, Taipei 110, Taiwan; 11 School of Health Care Administration, Taipei Medical University, Taipei 110, Taiwan

**Keywords:** COVID-19, artificial intelligence, machine learning, bibliometric analysis, health, coronavirus

## Abstract

The application of artificial intelligence (AI) to health has increased, including to COVID-19. This study aimed to provide a clear overview of COVID-19-related AI publication trends using longitudinal bibliometric analysis. A systematic literature search was conducted on the Web of Science for English language peer-reviewed articles related to AI application to COVID-19. A search strategy was developed to collect relevant articles and extracted bibliographic information (e.g., country, research area, sources, and author). VOSviewer (Leiden University) and Bibliometrix (R package) were used to visualize the co-occurrence networks of authors, sources, countries, institutions, global collaborations, citations, co-citations, and keywords. We included 729 research articles on the application of AI to COVID-19 published between 2020 and 2021. *PLOS One* (33/729, 4.52%), *Chaos Solution Fractals* (29/729, 3.97%), and *Journal of Medical Internet Research* (29/729, 3.97%) were the most common journals publishing these articles. The Republic of China (190/729, 26.06%), the USA (173/729, 23.73%), and India (92/729, 12.62%) were the most prolific countries of origin. The *Huazhong University of Science and Technology*, *Wuhan University*, and *the Chinese Academy of Sciences* were the most productive institutions. This is the first study to show a comprehensive picture of the global efforts to address COVID-19 using AI. The findings of this study also provide insights and research directions for academic researchers, policymakers, and healthcare practitioners who wish to collaborate in these domains in the future.

## 1. Introduction

The application of artificial intelligence (AI) to healthcare has increased rapidly [1]. AI involves the development of sophisticated algorithms to execute complex tasks efficiently and effectively. The main objective of applying AI to healthcare is to unfold hidden information from big data and assist healthcare policymakers and clinicians in making effective clinical decisions [2]. However, the application of AI technology to disease detection, cancer patient screening, therapy selection, reducing medication errors, and productivity improvement is now growing [3,4,5,6].

Furthermore, AI application to COVID-19 research has increased, especially to the diagnosis, classification, detection, severity, and mortality risk [7,8,9]. AI technology has already shown its potentiality to track the spread of coronavirus, as well as stratifying high-risk patients. It has also shown great effectiveness in predicting real-time infection rates by adequately analyzing the previous data [10]. Bibliometric analysis is a quantitative analysis of academic literature to describe the trends in publications, the contributions of authors and journals, countries’ productivity, and information about research cooperations and collaborations [11,12,13]. Bibliometric analysis can help to monitor the trends and patterns of effective literature in various areas, including healthcare [14].

In this study, we conducted bibliometric analysis and network visualization to provide a complete overview of the research trends, research domains, publication patterns, emerging topics, and global collaborations in the field of AI on COVID-19. This is the first study to quantitatively analyze the application of AI and hot areas of COIVD-19 research. Our study exposes the contribution of scientific knowledge by pointing out the gaps and providing a meaningful direction for future research of AI on COVID-19.

## 2. Materials and Methods

### 2.1. Search Strategy

We systematically searched articles in the Web of Science (WOS) between 1 February 2020 and 1 February 2021. Key search terms included *COVID-19 diagnosis, detection, classification, risk factors, severity, mortality, hospital stay, vaccine development, drug repurposing, epidemic trend, artificial intelligence, machine learning, deep learning, convolutional neural network, neural network, logistic regression, random forest, support vector machine,* etc. (Supplementary Table S1). Two authors also checked the reference lists of the included studies to identify additional relevant studies. As WOS does not include any preprints (non-peer-reviewed articles), studies published in Medrxiv, Arxiv, and Biorxiv were not included in our search.

### 2.2. Inclusion and Exclusion Criteria

We included all relevant articles that described the application of AI to address COVID-19. Conference proceedings and early access articles were included in our study. We excluded studies if they were published as reviews, books, editorials, letters, and conference abstracts. Furthermore, articles not published in the English language were also excluded.

### 2.3. Data Extraction

We retrieved data from WOS as a bibliographic information file (.bib file). The data exported included: (a) authors, (b) abstracts, (c) addresses, (d) ISSNs/ISBNs, (e) IDS numbers, (f) funding information, (g) PubMed IDs, (h) titles, (i) cited references, (j) times cited, (k) cited reference counts, (l) languages, (m) sources, (n) document types, (o) keywords, (p) source abbreviations, (q) author identifiers, (r) conference information, (s) publisher information, (t) research areas, (u) usage counts, and (v) highly cited.

### 2.4. Bibliometric Analysis

Bibliometric analysis helps to provide a deep summary of the recent trends in scientific publications. In this study, we presented publication patterns (top 10 productive countries and journals), publish domains, research activities (top keywords for coronavirus disease, technology, research focus, and data), author contributions, global cooperations, and co-citing references of AI research on COVID-19. The VOSviewer software (Rapenburg 70, 2311 EZ Leiden, Netherlands) (http://www.vosviewer.com/, accessed on 3 February 2021) was utilized to present the co-occurrence of authors’ keywords, authors’ contributions, global collaborations, and reference co-citation analyses. We also used the Bibliometrix R package (https://www.bibliometrix.org/, accessed on 3 February 2021) to calculate the frequency, percentage, and citations of each journal and country. A global collaboration map and other visualizations (corresponding information, author cooperation) were done by “Biblioshiny”.

## 3. Results

### 3.1. Literature Outputs

The literature search of Web of Science (WOS) yielded 1697 articles. We excluded 127 articles published as review articles, letters, editorial materials, meeting abstracts, corrections, and retracted publications. Furthermore, we excluded 13 articles published in a non-English language (Spanish, France, Hungarian, Italian, Russian, and German). After screening all titles and abstracts, eight-hundred twenty-eight articles were excluded due to a lack of adherence to the inclusion criteria, and seven-hundred twenty-nine articles were included in our study (Supplementary Figure S1).

### 3.2. Publication Patterns

Table 1 shows the distribution of articles in the top 10 countries. About 26.06% (190/729) of the articles were published in the Republic of China. The United States of America was the second highest country (173/729, 23.73%), followed by India (92/729, 12.62%). The number of citations was higher in the papers published from China and the USA, followed by Canada and Italy.

Two-hundred ninety-eight journals published 729 research articles. As shown in Table 2, *PLOS One* (*n* = 33, 4.52%), *Chaos Solution Fractals* (*n* = 29, 3.97%), and *Journal of Medical Internet Research* (*n* = 29, 3.97%) were the three journals that published a higher number of articles; followed by *IEEE Access* (27, 3.70%) and *Applied Intelligence* (*n* = 21, 2.88%). However, the *International Journal of Environmental Research and Public Health* (*n* = 221, 28.36%) had higher citations, followed by *Chaos Solution Fractals* (*n* = 201, 25.80%).

Table 3 shows the top 10 research areas, which published papers on AI and COVID-19. The research areas of *computer science artificial intelligence* (*n* = 85, 11.66%), *computer science information systems* (*n* = 77, 10.56%), and *multidisciplinary science* (*n* = 75, 10.28%) were the top ones on the application of AI to COVID-19. However, *radiology nuclear medicine imaging*, and *computer science information systems* had higher citations than the other research areas.

### 3.3. Research Activity

Table 4 presents the authors’ keywords used in the study of AI and COVID-19. It is divided into four groups: (1) disease (2) technology (3) types of data, and (4) research focus. In the disease group, COVID-19 was the most common keyword, followed by SARS-CoV-2 and coronavirus. Among all the keywords provided by authors on technology research, the top 5 technologies were deep learning, machine learning, artificial intelligence, convolutional neural networks, and transfer learning. The application of AI to COVID-19 was mainly focused on pandemics, prediction, classification, and diagnoses.

**Figure 1:**
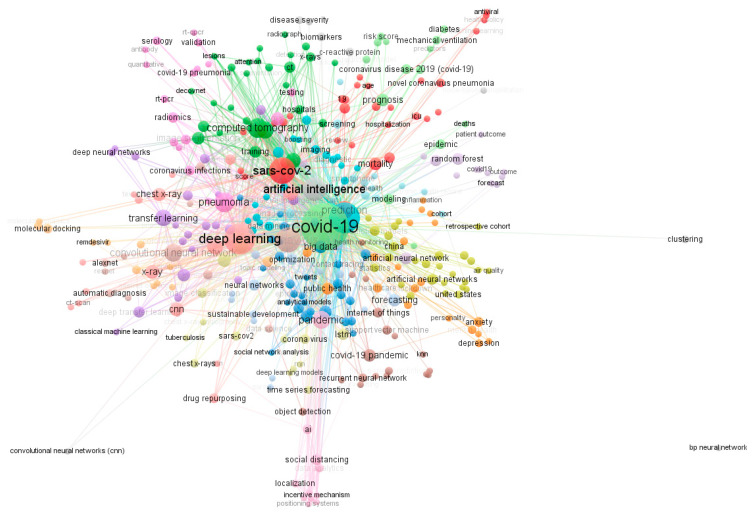
The co-occurrence of authors’ keywords. There were 1824 keywords found in the included studies. The thickness of the lines shows the strength of the association between keywords. The thickness was determined by the frequency of keywords in the included studies.

VOSviewer classified 1824 keywords into 22 clusters, as shown in Figure 1. The strengths of the association of COVID-19, deep learning, machine learning, SARS-CoV-2, and coronavirus were 1429, 559, 515, 300, and 286.

**Figure 2:**
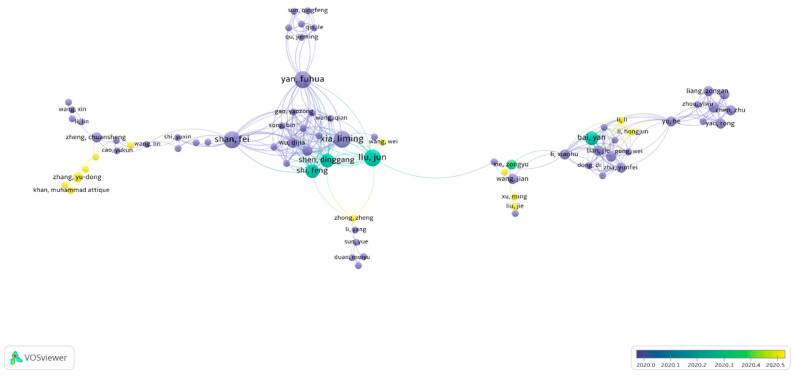
The global network of coauthors.

Author contribution and global cooperation: We applied a cut-off of two papers per author and presented the global cooperation of authors visually. Among 4233 authors, Figure 2 shows the cooperation of 315 authors globally. Nineteen authors had a strong collaboration with others and published at least four papers together. The thickness of the line shows the association among the authors, and the size of the circle shows the number of articles published together. For example, the authors Yan, Fuhua and Shan, Fei published more papers together than others.

**Figure 3:**
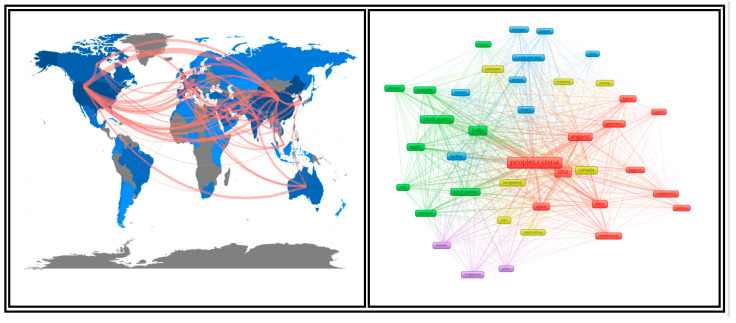
The global network of the 39 countries (at least five papers). Nota bene (N.B.): There were 82 countries’ researchers who conducted collaborative AI research on COVID-19.

Figure 3 shows the visual network of the 39 countries that contributed a least five papers together. It also presents the strength of their partnership. For example, the Republic of China had a strong collaboration with the USA, Italy, Canada, Australia, India, and South Korea. India had a strong collaboration with England, Australia, South Korea, China, and Spain. Russia had a strong collaboration with England, Egypt, Turkey, and Saudi Arabia.

**Figure 4:**
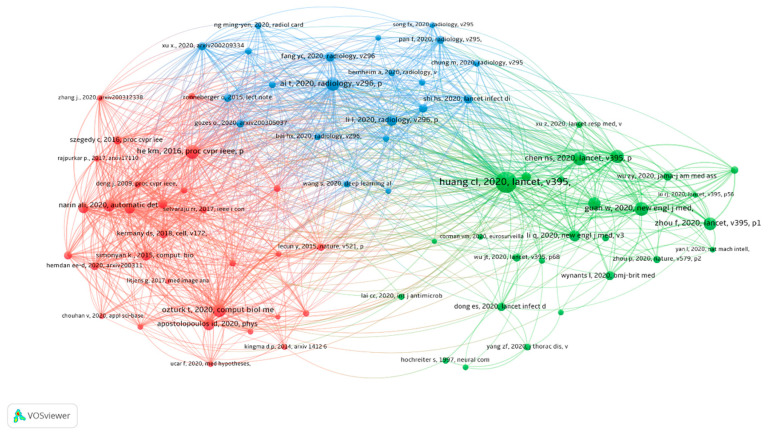
The co-citation network of references on AI and COVID-19.

References co-citation analysis: In the co-citation analysis, we assessed all the cited references in the AI field. We identified 729 articles related to AI and COVID-19, with 20,959 articles cited, with an average of 29 references per article. The top 10 most frequently cited articles are presented in Table 5. The most cited paper was from Huang Chaolin et al. 2020, which was published in Lancet and cited 150 AI-related papers. We selected 78 references on AI and COVID-19 that were cited at least 20 times and present them in Figure 4. There are three clusters marked with different colors. The first cluster is in red color and contains 30 references, the second cluster in green containing 28 references, and the third cluster in blue containing 21 references.

## 4. Discussion

To our knowledge, this is the first intensive bibliometric study of scientific articles on the application of AI to address COVID-19. This study shows publication patterns, author cooperations, global collaborations, and research hotspot trends. The Republic of China was more prolific in publishing papers on AI and COVID-19, followed by the USA and India. *PLOS One* published a higher number of AI articles than *Chaos Solution Fractals* and *Journal of Medical Internet Research*. Most of the researchers published their articles in the area of *computer science artificial intelligence, computer science information system, and multidisciplinary sciences*. Furthermore, the Republic of China had a strong collaboration with the USA, Italy, Canada, Australia, and India.

### 4.1. Global Trends of AI Research on COVID-19

Since the number of COVID-19 cases has increased rapidly and it appeared as a lethal global pandemic; researchers from all over the world have been trying to focus on COVID-19 research and to tackle the situation using AI [15,16]. The main purposes of their research are to develop and validate potential models to diagnose, detect, and stratify COVID-19 patients quickly. Moreover, calculating epidemic trends, the identification of biomarkers, finding potential drugs, and mortality risk are the areas of interest [17,18,19,20]. There were over 700 AI publications focused on different areas of COVID-19.

For journal sources, the top 10 journals published approximately 30 percent (214/729) of the total publications. Among them, *PLOS One*, *Chaos Solution Fractals*, and *Journal of Medical Internet Research* were more productive than the other journals. More interestingly, the Radiology journal published only five articles, but had higher citations. Moreover, *Journal of Thoracic Disease* and *IEEE Transactions on Medical Imaging* were not in the top 10 most prolific journals lists, but in the top 10 journal citations lists. This is because researchers are more focused on image analysis research to screen COVID-19 patients more accurately and rapidly. Journals publishing AI research were almost all in the health domain, with more focus, as expected, on the fields of *computer science, artificial intelligence*, and *computer science information systems*.

### 4.2. The Coauthorship Networks of AI Research on COVID-19

AI is becoming more popular in the healthcare industry due to its ability to solve complex disease patterns, for earlier identification of risk, and for personalized treatment [21,22]. As an AI-based model can handle a huge amount of patient data and recognize patterns, AI-based technology has been utilized as a powerful solution for addressing the COVID-19 pandemic [10,23]. Previous studies reported getting the utmost benefits from AI, research collaborations for global consideration via data sharing, and technological supports [1,24,25]. Our study showed that the Republic of China collaborated with more countries on AI-related COVID-19 research (Supplementary figures). In China, *Huazhong University of Science and Technology* and *Wuhan University* and, in Saudi Arabia, *King Saud University* collaborated on more research with other institutions (Supplementary figures). We identified 4233 authors who were dedicated to publishing papers on AI and COVID-19. Of those authors, only seven authors published at least five papers, and eleven authors published at least four papers. While searching the areas of research interest, most of the authors focused on COVID-19 diagnosis, detection, and classification.

### 4.3. Limitations

Our study had several limitations. First, we only included studies published in English. Secondly, we did not include studies published in the Rxiv repository. We might have missed many studies related to AI and COVID-19; however, they were not fully peer-reviewed articles. Third, we only extracted and analyzed data from WOS; although WOS is a large database that offers a wide variety of research and includes all SCI and SSCI listed journal. Scopus and PubMed data will be used in a future study.

## 5. Conclusions

Literature in the field of AI on COVID-19 represented over 700 publications. This bibliometric analysis depicted a comprehensive overview of current research trends of AI application to COVID-19. The findings of this study also showed that the focus area of AI research mainly was on COVID-19 diagnosis, detection, epidemic trends, classification, and drug repurposing. Indeed, high-income countries such as the USA, China, Italy, and Spain conducted more research on the application of AI to COVID-19. The efficiency and diversity of applications of AI (e.g., machine learning and deep learning) to patient screening, early treatments, and improving patient care are already visible, and the further implementation of AI in real-world clinical practice is expected to increase, which ultimately will help to address any pandemic such as COVID-19.

## Figures and Tables

**Table 1:** The distribution of AI articles in top 10 countries (by quantity).

Countries	Ranking Based on Total Output	Output, *n* (%)	Ranking Based on Citations	Citations, *n* (%)	Strength
China	1	190 (26.06)	1	1275 (31.52)	79,056
USA	2	173 (23.73)	2	637 (15.75)	61,714
India	3	92 (12.62)	6	323 (7.98)	39,757
Italy	4	55 (7.54)	4	332 (8.20)	22,338
England	5	54 (7.40)	9	321 (7.93)	25,759
Saudi Arabia	6	44 (6.03)	8	91 (2.25)	18,945
Canada	7	42 (5.76)	3	547 (13.52)	14,136
South Korea	8	37 (4.07)	7	176 (4.35)	18,482
Spain	9	33 (4.52)	10	53 (1.31)	16,735
Turkey	10	31 (4.25)	5	289 (7.14)	17,613

**Table 2:** The distribution of AI articles in the top 10 journals (by quantity).

Journal Names	Ranking Based on Total Output	Output, *n* (%)	Ranking Based on Citations	Citations, *n* (%)	Strength
PLOS One	1	33 (4.52)	4	73 (9.37)	4711
Chaos Solution Fractals	2	29 (3.97)	2	201 (25.80)	3137
Journal of Medical Internet Research	3	29 (3.97)	6	49 (6.29)	2967
IEEE Access	4	27 (3.70)	3	93 (11.93)	5207
Applied Intelligence	5	21 (2.88)	9	12 (1.54)	5565
CMC Computers Materials Continua	6	19 (2.60)	5	70 (8.98)	2839
Scientific Reports	7	17 (2.33)	9	15 (1.92)	2301
International Journal of Environmental Research and Public Health	8	15 (2.05)	1	221 (28.36)	788
Applied Sciences Basel	9	13 (1.78)	7	29 (3.72)	1282
Applied Soft Computing	10	11 (5.14)	8	16 (2.05)	2909

**Table 3:** The distribution of AI articles in the top 10 areas (by quantity).

Research Area	Ranking Based on Total Output	Output, *n* (%)	Ranking Based on Citations	Citations, *n* (%)
Computer Science Artificial Intelligence	1	85 (11.66)	4	173 (5.54)
Computer Science Information Systems	2	77 (10.56)	2	196 (6.28)
Multidisciplinary Sciences	3	75 (10.28)	6	153 (4.90)
Electrical and Electronics Engineering	4	67 (9.19)	3	227 (7.28)
Computer Science Interdisciplinary Applications	5	63 (8.64)	9	623 (19.98)
Medical Informatics	6	63 (8.64)	5	211 (6.76)
Health Care Sciences Services	7	55 (7.54)	9	190 (6.09)
Radiology Nuclear Medicine Imaging	8	53 (7.27)	1	540 (17.71)
Biomedical Engineering	9	45 (6.17)	7	497 (15.93)
Public Environmental Occupational Health	10	45 (6.17)	8	308 (9.87)

**Table 4:** The top keywords of AI in COVID-19 publications.

Category	Number of Authors’ Keywords, *n* (%)
Disease	
COVID-19	461 (25.27)
SARS-CoV-2	89 (4.87)
Coronavirus	75 (4.11)
Research technology	
Deep learning	137 (7.51)
Machine learning	135 (7.40)
Artificial intelligence	67 (3.67)
Convolutional neural networks	30 (1.64)
Transfer learning	25 (1.37)
Research data	
Computed tomography	41 (2.24)
X-ray	16 (0.87)
Big data	12 (0.65)
Social media	11 (0.60)
Twitter	10 (0.54)
Research focus	
Pandemic	30 (1.64)
Prediction	29 (1.58)
Classification	22 (1.20)
Diagnosis	13 (0.71)
Mortality	12 (0.65)
Prognosis	12 (0.65)
Forecasting	12 (0.65)
Severity	8 (0.43)
Risk factor	6 (0.32)

**Table 5:** The top 10 cited references on AI to address COVID-19.

**Rank**	**Author**	**Journal**	**Citations, *n***	**Strength**
1	Huang Chaolin et al. 2020	Lancet	150	905
2	Wei-jie Guan et al. 2020	The New England Journal of Medicine	81	403
3	Kaiming He et al. 2016	IEEE Conference on Computer Vision and Pattern Recognition	78	688
4	Tao Ai et al. 2020	Radiology	75	616
5	Nanshan Chen et al. 2020	Lancet	75	460
6	Dawei Wang et al. 2020	JAMA: The Journal of the American Medical Association	74	437
7	Fei Zhou et al. 2020	Lancet	71	269
8	Tulin Ozturk et al. 2020	Computers in Biology and Medicine	68	539
9	Na Zhu et al. 2020	The New England Journal of Medicine	64	320
10	Ioannis D Apostolopoulos et al. 2020	Physical and Engineering Sciences in Medicine	61	519

## Supplementary Materials

The following are available online at https://www.mdpi.com/article/10.3390/healthcare9040441/s1, Table S1: Search strategy, Figure S1: Study selection, Figure S2= Distribution of countries’ contribution, Figure S3: Distribution of journal’s contribution, Figure S4: China’s collaboration with other countries, Figure S5: Distribution of institute’s contribution, Figure S6: Distribution of Huazhong University of Science and Technology collaboration.

## References

[1] He J., Baxter S.L., Xu J., Xu J., Zhou X., Zhang K. (2019). The practical implementation of artificial intelligence technologies in medicine. Nat. Med..

[2] Murdoch T.B., Detsky A.S. (2013). The inevitable application of big data to health care. JAMA.

[3] Jiang F., Jiang Y., Zhi H., Dong Y., Li H., Ma S., Wang Y., Dong Q., Shen H., Wang Y. (2017). Artificial intelligence in healthcare: Past, present and future. Stroke Vasc. Neurol..

[4] Islam M.M., Yang H.-C., Poly T.N., Jian W.-S., Li Y.-C.J. (2020). Deep learning algorithms for detection of diabetic retinopathy in retinal fundus photographs: A systematic review and meta-analysis. Comput. Methods Programs Biomed..

[5] Islam M., Poly T.N., Walther B.A., Yang H.C., Li Y.-C.J. (2020). Artificial intelligence in ophthalmology: A meta-analysis of deep learning models for retinal vessels segmentation. J. Clin. Med..

[6] Poly T.N., Islam M.M., Muhtar M.S., Yang H.-C., Nguyen P.A.A., Li Y.-C.J. (2020). Machine learning approach to reduce alert fatigue using a disease medication–related clinical decision support system: Model development and validation. JMIR Med. Inform..

[7] Nguyen T.T. (2020). Artificial intelligence in the battle against coronavirus (COVID-19): A survey and future research directions. arXiv.

[8] Nan S.N., Ya Y., Ling T.L., Nv G.H., Ying P.H., Bin J. (2020). A prediction model based on machine learning for diagnosing the early COVID-19 patients. medRxiv.

[9] Ghaderzadeh M., Asadi F. (2020). Deep learning in detection and diagnosis of Covid-19 using radiology modalities: A systematic review. arXiv.

[10] Ahuja A.S., Reddy V.P., Marques O. (2020). Artificial Intelligence and COVID-19: A Multidisciplinary Approach. Integr. Med. Res..

[11] Guler A.T., Waaijer C.J., Palmblad M. (2016). Scientific workflows for bibliometrics. Scientometrics.

[12] Ahmadvand A., Kavanagh D., Clark M., Drennan J., Nissen L. (2019). Trends and visibility of “digital health” as a keyword in articles by JMIR publications in the new millennium: Bibliographic-bibliometric analysis. J. Med Internet Res..

[13] Taj F., Klein M.C., van Halteren A. (2019). Digital health behavior change technology: Bibliometric and scoping review of two decades of research. JMIR mHealth uHealth.

[14] Peng C., He M., Cutrona S.L., Kiefe C.I., Liu F., Wang Z. (2020). Theme trends and knowledge structure on mobile health apps: Bibliometric analysis. JMIR mHealth uHealth.

[15] Vaishya R., Javaid M., Khan I.H., Haleem A. (2020). Artificial Intelligence (AI) applications for COVID-19 pandemic. Diabetes Metab. Syndr. Clin. Res. Rev..

[16] Salman F.M., Abu-Naser S.S., Alajrami E., Abu-Nasser B.S., Alashqar B.A. (2020). Covid-19 Detection Using Artificial Intelligence.

[17] Jamshidi M., Lalbakhsh A., Talla J., Peroutka Z., Hadjilooei F., Lalbakhsh P., Jamshidi M., La Spada L., Mirmozafari M., Dehghani M. (2020). Artificial intelligence and COVID-19: Deep learning approaches for diagnosis and treatment. IEEE Access.

[18] Naudé W. (2020). Artificial intelligence vs. COVID-19: Limitations, constraints and pitfalls. AI Soc..

[19] Jin C., Chen W., Cao Y., Xu Z., Tan Z., Zhang X., Deng L., Zheng C., Zhou J., Shi H. (2020). Development and evaluation of an artificial intelligence system for COVID-19 diagnosis. Nat. Commun..

[20] Zhou Y., Wang F., Tang J., Nussinov R., Cheng F. (2020). Artificial intelligence in COVID-19 drug repurposing. Lancet Digit. Health.

[21] Schork N.J. (2019). Artificial intelligence and personalized medicine. Precis. Med. Cancer Ther..

[22] Awwalu J., Garba A.G., Ghazvini A., Atuah R. (2015). Artificial intelligence in personalized medicine application of AI algorithms in solving personalized medicine problems. Int. J. Comput. Theory Eng..

[23] Lalmuanawma S., Hussain J., Chhakchhuak L. (2020). Applications of machine learning and artificial intelligence for Covid-19 (SARS-CoV-2) pandemic: A review. Chaos Solitons Fractals.

[24] Mikhaylov S.J., Esteve M., Campion A. (2018). Artificial intelligence for the public sector: Opportunities and challenges of cross-sector collaboration. Philos. Trans. R. Soc. A Math. Phys. Eng. Sci..

[25] Shao Z., Yuan S., Wang Y. (2020). Institutional collaboration and competition in artificial intelligence. IEEE Access.

