# Supplementary material for: Application of Artificial Intelligence in COVID-19 Pandemic: Bibliometric Analysis
Source: Healthcare (Basel). 2021 Apr 9;9(4):441. doi: 10.3390/healthcare9040441 (PMC8070493; doi:10.3390/healthcare9040441)
Supplement: Supplementary file 1 [file healthcare-09-00441-s001.pdf]

Supplementary Table:

Table S1:

**Keywords**
**Step 1**: "Artificial intel*", OR "machine learning" OR "deep learning" OR "neural network*", OR "CNN", OR "convolutional neural network" OR "SVM" OR "Random forest" OR "Logistic regression" OR "RNN" OR "LSTM"<br>Step 2: ("COVID-19" AND "risk fact*") OR ("COVID-19" AND "disease detection") OR ("COVID-19" AND "disease classif*") OR ("COVID-19" AND "disease severity") OR ("COVID-19 AND "mortality risk") OR ("COVID-19 AND "epidemic tren*")….<br>Step 1 + Step 2

__Supplementary figure__:

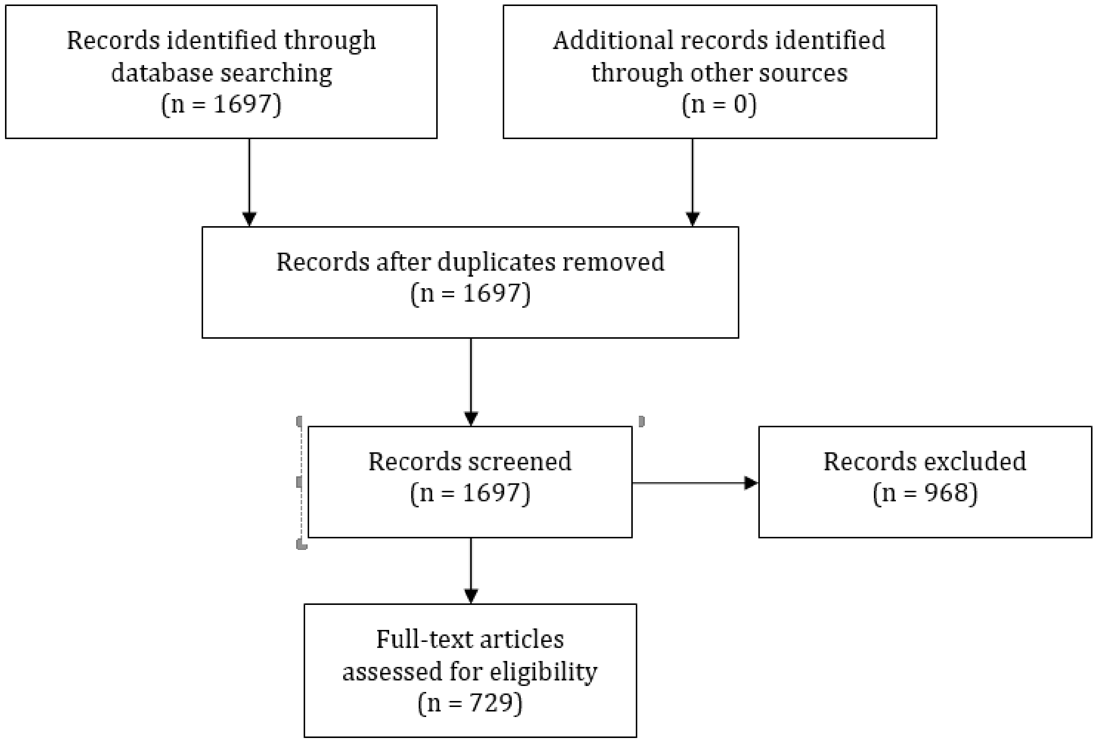

Figure S1. Study selection.

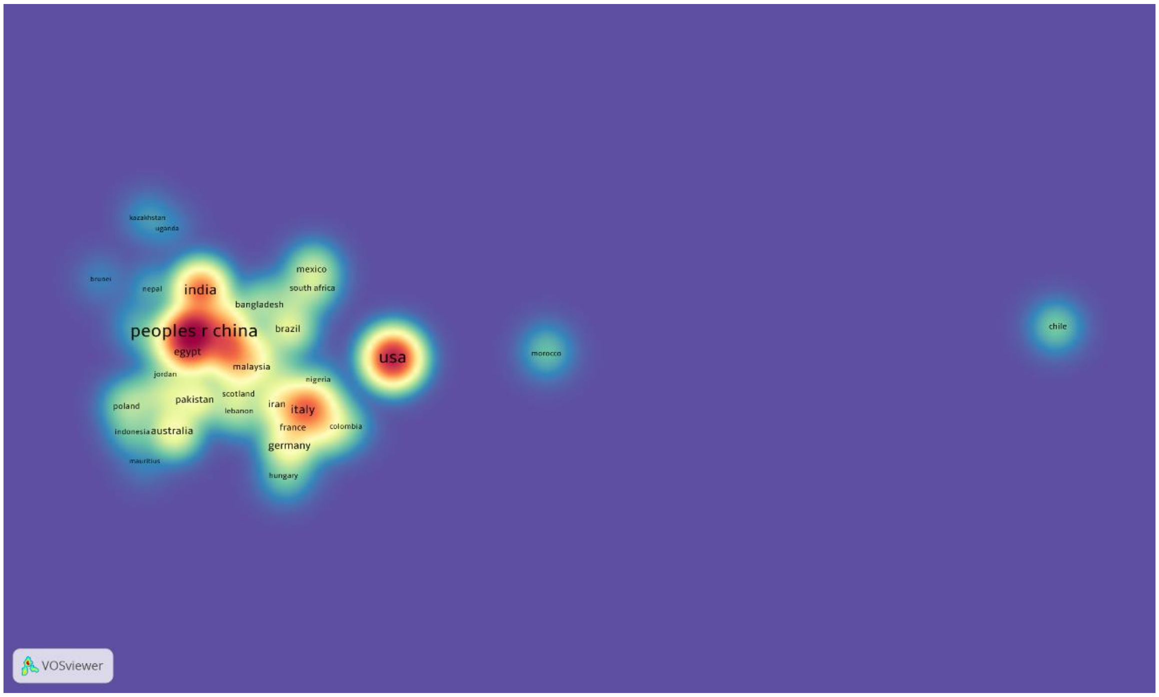

Figure S2: Distribution of countries' contribution.

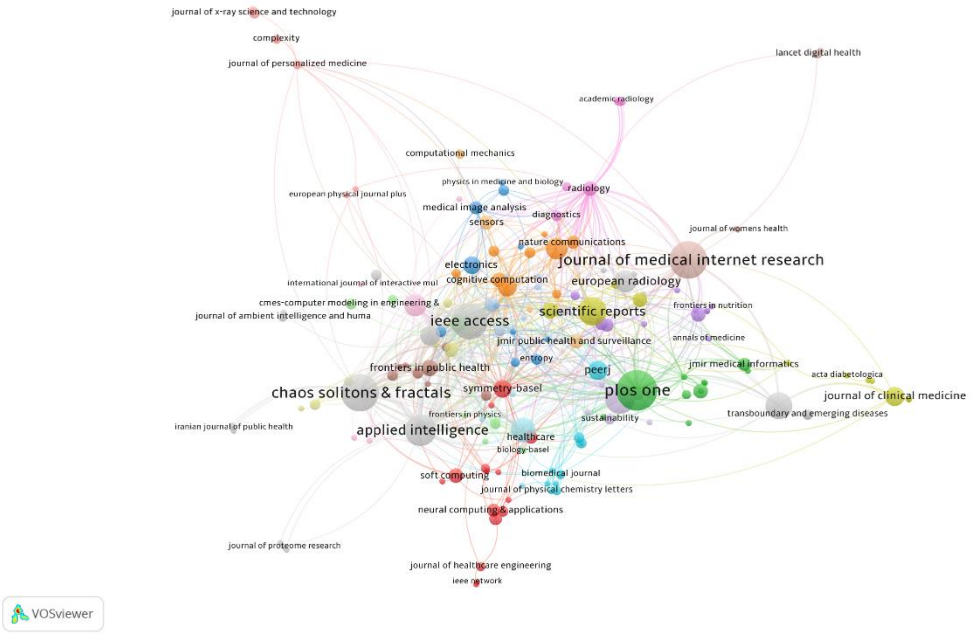

Figure S3: Distribution of journal's contribution.

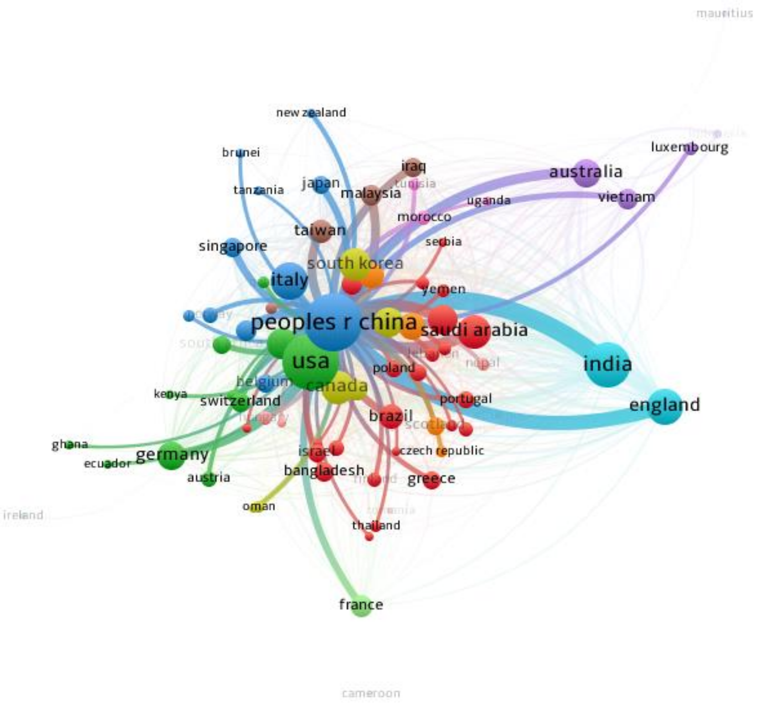

Figure S4: China's collaboration with other countries

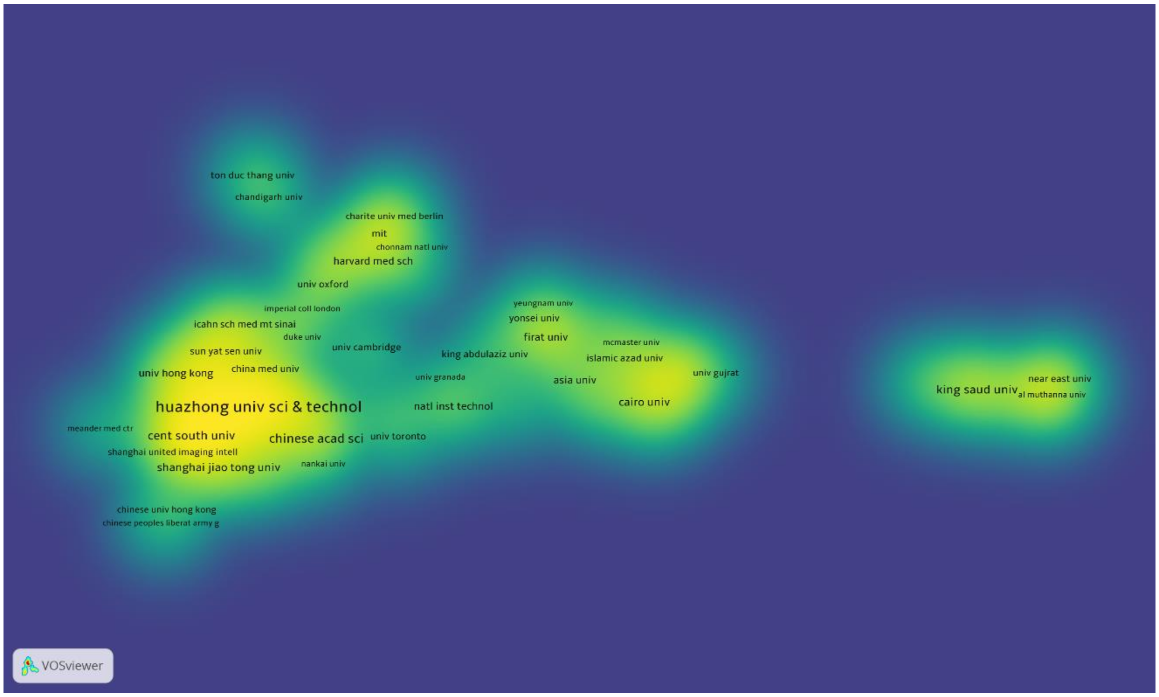

Figure S5: Distribution of institute's contribution.

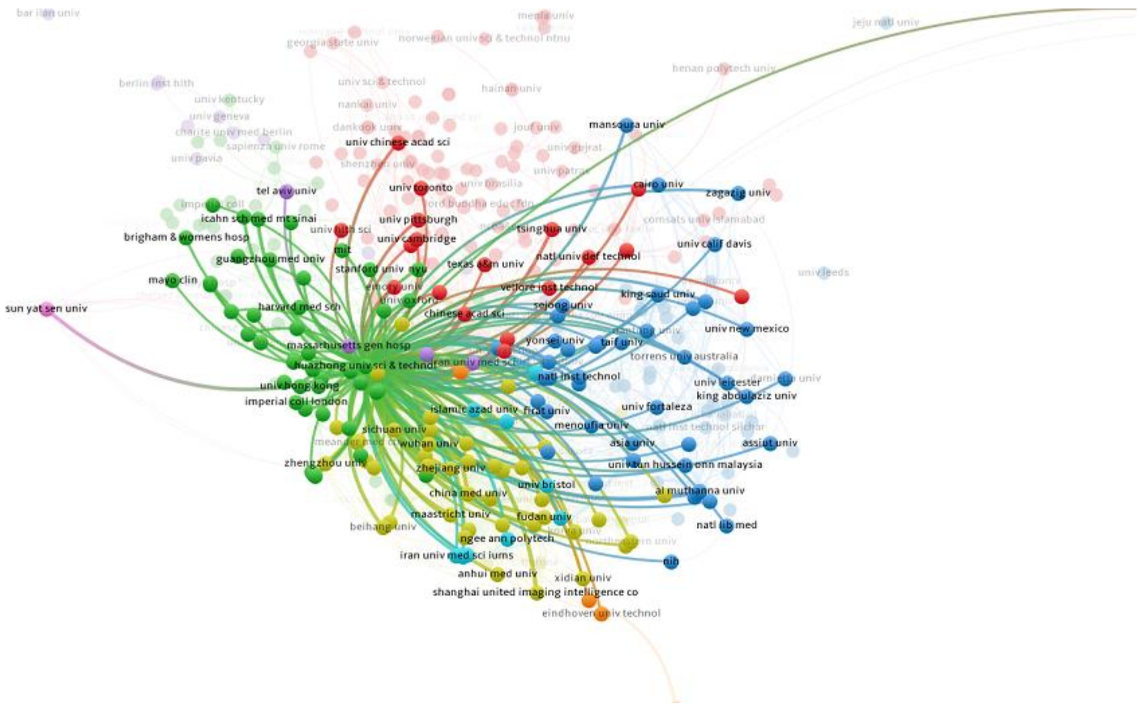

Figure S6: Distribution of Huazhong University of Science and Technology collaboration.
